# protGear: A protein microarray data pre-processing suite

**DOI:** 10.1016/j.csbj.2021.04.044

**Published:** 2021-04-24

**Authors:** Kennedy Mwai, Nelson Kibinge, James Tuju, Gathoni Kamuyu, Rinter Kimathi, James Mburu, Emily Chepsat, Lydia Nyamako, Timothy Chege, Irene Nkumama, Samson Kinyanjui, Eustasius Musenge, Faith Osier

**Affiliations:** a Epidemiology and Biostatistics Division, School of Public Health, University of the Witwatersrand, Johannesburg, South Africa; b Centre for Geographic Medicine Research (Coast), Kenya Medical Research Institute-Wellcome Trust Research Programme, Kilifi, Kenya; c Centre of Infectious Diseases, Heidelberg University Hospital, Heidelberg, Germany; d Department of Biotechnology and Biochemistry, Pwani University, Kilifi, Kenya; e Centre for Tropical Medicine and Global Health, Nuffield Department of Clinical Medicine, University of Oxford, Oxford, United Kingdom

**Keywords:** Protein microarray, Normalisation, Background correction, Batch correction, Reproducibility

## Abstract

Protein microarrays are versatile tools for high throughput study of the human proteome, but systematic and non-systematic sources of bias constrain optimal interpretation and the ultimate utility of the data. Published guidelines to limit technical variability whilst maintaining important biological variation favour DNA-based microarrays that often differ fundamentally in their experimental design. Rigorous tools to guide background correction, the quantification of within-sample variation, normalisation, and batch correction specifically for protein microarrays are limited, require extensive investigation and are not centrally accessible.

Here, we develop a generic one-stop-shop pre-processing suite for protein microarrays that is compatible with data from the major protein microarray scanners. Our graphical and tabular interfaces facilitate a detailed inspection of data and are coupled with supporting guidelines that enable users to select the most appropriate algorithms to systematically address bias arising in customized experiments. The localization and distribution of background signal intensities determine the optimal correction strategy. A novel function overcomes the limitations in the interpretation of the coefficient of variation when signal intensities are at the lower end of the detection threshold. We demonstrate essential considerations in the experimental design and their impact on a range of algorithms for normalization and minimization of batch effects.

Our user-friendly interactive web-based platform eliminates the need for prowess in programming. The open-source R interface includes illustrative examples, generates an auditable record, enables reproducibility, and can incorporate additional custom scripts through its online repository. This versatility will enhance its broad uptake in the infectious disease and vaccine development community.

## Graphical abstract

**Figure:**
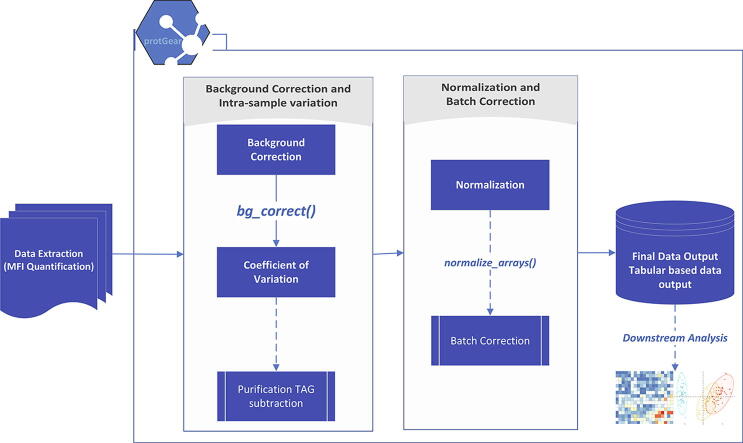

## 1 Introduction

Protein microarray technology is increasingly utilised for vaccine candidate discovery among other range of applications in the ‘omics era’ with hundreds to thousands of antigen-specific antibodies analysed simultaneously [1], [2], [3], [4], [5]. Antibody data are correlated with infection or disease outcomes in experimental models and observational studies [6]. The platform is also useful for the dissection of variant-specific antibodies induced by polymorphic proteins [7].

Although multiple pipelines of the analysis of DNA microarrays are published [8], [9], they are not always suitable for proteins because of fundamental differences in the underlying experimental design. In the former, gene expression levels are typically compared by mixing test and control samples that are labelled with a pair of distinct fluorescent dyes. The emission signal at a defined locus in a test sample is expressed as a ratio, relative to its counterpart in the control. Normalization in this context factors in intrinsic differences between dyes and the relative efficiency of their incorporation into the samples under investigation. In contrast, standard protein microarrays (the reverse and forward phase microarrays) quantify the absolute fluorescent emission detected following antibody binding to a single protein, therefore other considerations for normalization become more important.

Similarly, although the concordance between replicates rises with increasing signal intensity (mean–variance dependence) for both DNA and protein microarrays, the respective normalization algorithms differ. An expectation of minimal variation in the majority of genes with the exception of the one(s) under investigation is the norm in standard DNA microarray experiments [10]. The exact opposite is true for experimental designs where important differences in antibody binding between individuals and proteins underpin the hypothesis [3]. Consequently, while scaling down variation for DNA microarrays serves the correct purpose, some algorithms may mask important biological variation in responses to proteins [10].

Tools to guide the rigorous processing of protein microarray data are limited [11], [12], [13], [14], some are time-consuming to optimize and not centralized [15]. Some of the available tools are Protein array web explorer (PAWER) [12], Protein Microarray Database (PMD) [14], Protein Microarray Analyser (PMA) [15], Prospector, Protein Array Analyser (PAA) [11]. Here, we provide a one-stop data-processing suite that empowers users to determine the most appropriate method for each data-handling step by comparing the different data handling techniques. A detailed comparison of the tools is documented in Supplementary-D. We systematically address background correction [16] within-sample variation [10], normalisation [17] and batch correction [18]. Our easy-to-use interactive web-based R interface [19] and illustrative examples enable wide utility.

## 2 Methods

**Fig. 1:**
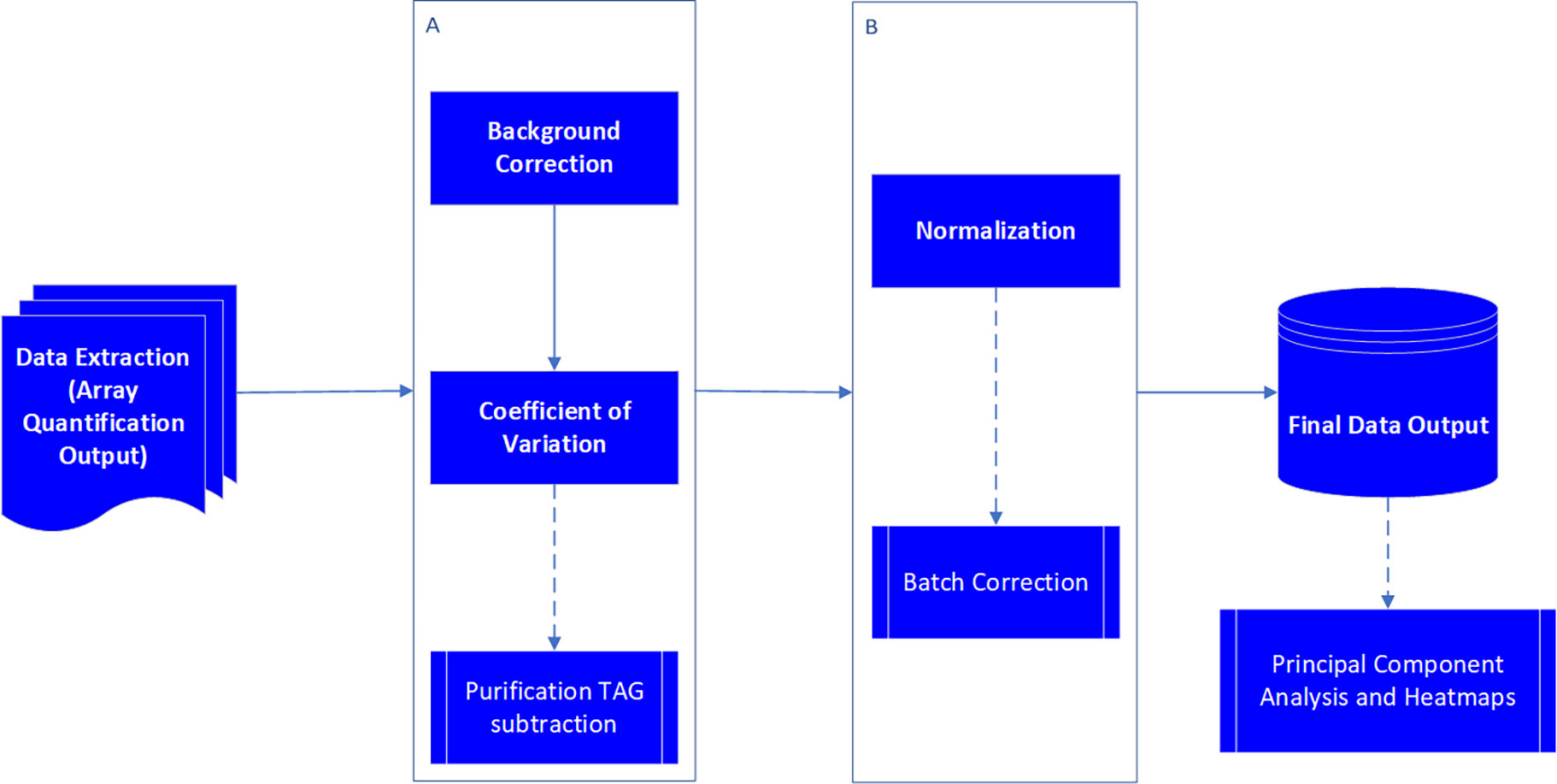
protGear data processing scheme. Dotted lines indicate optional steps. Tag subtraction is applied for antigens containing purification tags. Batch correction is relevant when multiple samples from the same sample set are processed in more than one experiment.

The data processing suite incorporates a range of sequential statistical functions organized into an R package with accompanying guidance notes (Fig. 1).

We examine the performance of protGear using data from KILchip v1.0, a protein microarray chip designed to enable the simultaneous detection of antibodies against > 100 proteins in large cohort studies. *Plasmodium falciparum* proteins were printed in triplicate on a slide divided into mini-arrays, defined as the region allocated to a discrete sample and can be further divided into blocks [3].

### 2.1 Data extraction

Microarray image analysis software captures multiple parameters in relation to antibody intensity in a pre-prepared template mostly referred to as a “*.gal”* file. [Supplementary A]. Quantification softwares for estimating pixel intensities for example Proscanarray Express software (PerkinElmer) [1], QuantArray software (GSI Lumonics) and GenePix® Pro software (Molecular Devices) [20] generate data with similar parameters. Pixel intensities for each spot are typically reported as means or medians with respective standard deviations. We recommend the median as it is relatively insensitive to outliers. We created a versatile tool that can be adapted to load data output format from different quantification softwares mentioned above and this is highlighted in Supplementary A.

### 2.2 Background correction

Background intensity is the signal emitted by sources other than the sample under investigation [16], [21]. In microarrays, it arises either from the glass slide and/or from the non-specific binding of analytes and can vary within and between slides [10], [16], [21]. The foreground is the total spot intensity and includes the background.

**Fig. 2:**
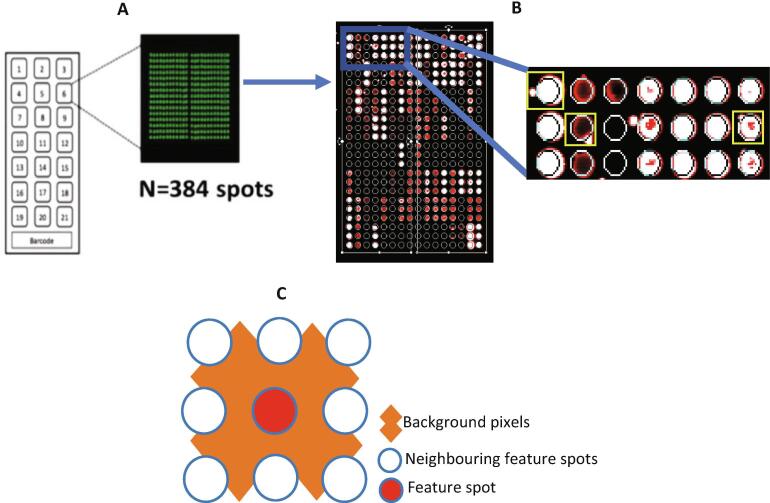
Background correction: artefacts add noise to the signal intensity A) A microarray slide with 21 mini arrays and a barcode. Each mini array has a specific number of features represented by a spot. B) Artefacts [spots surrounded with yellow boxes] Kamuyu 2018. C) The total foreground intensity associated with feature spot typically includes the local background. (For interpretation of the references to colour in this figure legend, the reader is referred to the web version of this article.)

The background is typically calculated using a circular region around the spot (Fig. 2). In GenePix® for example, this is estimated using a diameter three times that of the corresponding spot indicator [20]. We adapted subtraction and model-based functions for background correction using a combination of the GenePix® Pro [20] and Linear Models for and Microarray Data (Limma) [8)] with minor modifications respectively. When the background exceeds the foreground, we implement functions to enable mathematical computation as explained under moving minimum background and half moving minimum background correction [16], [22]. Prior to background correction, it is important to visually inspect the protein microarray data for any spatial biases. protGear provides two functionalities to visually inspect the slides, however, we recommend inspection of the scanned images [Fig. 2] since the data file might not record all the spatial artefacts. The dashboard includes a function to visualize the printing buffer spots which are used to monitor any background reactivity and detection of any potential protein carry over during printing [3].

protGear provides a range of background subtraction methods based on understanding the distribution of the artefacts in specific experiments and supported with graphical outputs. The *local background* is the signal detected in the immediate vicinity of a spot. Subtracting the median local background pixel intensities from the foreground is thought to give an unbiased estimator of the true signal intensity for that specific spot [16]. However, uneven variation in the local background across an entire slide may skew the data [23] and can be minimized by implementing a *global background* subtraction. This subtracts the median of all the local background intensities for a given slide from each spot on that slide. When artefacts are localized to a specific region of a slide often referred to as a block or mini-array, the *moving minimum background* or *half moving minimum background correction* options can be adopted [8]. The former restricts the subtraction of local backgrounds to the block within the slide. Since many spots may be affected within a block, it utilizes the minimum rather than the median local background. Zero or negative values are subsequently set to half the minimum of the positive corrected background intensities different from limma implementation that sets any intensity which is <0.5 after background subtraction to be equal to 0.5.

The *normal and exponential model (normexp)* method is recommended when the distribution of background intensities is normal, but that of the background-corrected data is exponential [24]. The background intensities are fitted as covariates in a convolution model and the expected signal given the observed foreground becomes the corrected intensity. This yields a smooth monotonic function of positive background-corrected intensities and replaces negative values on the entire slide with a single positive co-efficient [25].

The log*-linear background correction* method utilizes the range of positive background-corrected values on the slide to compute a log-linear smooth monotonic function from which negative values are interpolated [22]. It can be considered for data that do not fit the above distributions.

### 2.3 Coefficient of variation.

Technical replicates assess within-sample variability and help to quantify the reliability of the experimental procedures. They can minimize data loss when for example a single spot performs poorly, and others succeed. They are also utilized to detect sample-specific experimental variability linked to outliers or caused by specific reagents. Although many replicates are advantageous, this comes at a cost and may reduce experimental throughput.

A function to evaluate the extent of within-sample variability is implemented using the Coefficient of Variation (CV). This is a measure of accuracy expressed defined as; $\mathrm{CV}\%=\left(\frac{\sigma }{\overset{¯}{x}}\right)\times 100$, where σ is the standard deviation divided by the mean $\overset{¯}{x}$, and expressed as a percentage [10]. Users can define a threshold (cut-off) for the CV using *n*-replicates and visualize those that do not meet this criterion. Replicate spots that are acceptable are averaged [26]. Additional functions enable the visualization and subtraction of signal intensities from purification tags, where applicable. Protein purification tags refer to specific amino acids or polypeptides fused to target proteins to facilitate their subsequent affinity purification. In the case of the datasets used here, the tags include the CD4 *hexa*-histidine, Maltose binding protein (MBP) and Glutathione S-transferase (GST) tags [3]. Purification-tag is specific to different experiments and designs, purification-tag subtraction step is optional as shown in Fig. 1.

### 2.4 Normalisation.

Data normalization minimizes the mean–variance dependence (MVD) that is common in microarray experiments and may mask true biological variability, Fig. 5A [9], [27]. Normalisation transforms the intensities to a scale where the variance, $Var\left(\mathrm{MFI}\right)$ is independent of the mean, $E\left(\mathrm{MFI}\right)$ where MFI is the (Mean Fluorescent Intensity). Although many normalisation strategies have been proposed [28] we focus on five techniques applicable to protein microarrays.

#### 2.4.1 Log_2_ normalisation

Log transformations reduce the bias in variance between high and low values by converting the data from a multiplicative to an additive distribution. Wider variance is typically observed for higher signal intensities. Log_2_ transformation is readily compatible with the doubling sample dilutions typically employed on the bench. The background-corrected values $\overset{⌢}{\underset{\mathrm{ijkr}}{y}}=y_{\mathrm{ijkr}}-\overset{⌢}{\alpha }$; where $\overset{⌢}{\alpha }$ is the estimate of the median background level and $y_{\mathrm{ijkr}}$ is the estimated intensity for a spot r of protein feature k in the mini-array j on the $i^{\mathrm{th}}$ slide. The transformation is defined as $\log_{2}\left(\overset{⌢}{\underset{\mathrm{ijkr}}{y}}\right)=\log_{2}\left(y_{\mathrm{ijkr}}-\overset{⌢}{\alpha }\right)$. However,$\log_{2}\left(\overset{⌢}{\underset{\mathrm{ijkr}}{y}}\right)$ is not defined for $y_{\mathrm{ijkr}}\leq \overset{⌢}{\alpha }$ or for intensities where $y_{\mathrm{ijkr}}-\overset{⌢}{\alpha }<0$. Additionally, the asymptotic variance of $\log\left(\overset{⌢}{\underset{\mathrm{ijkr}}{y}}\right)$ is approximately constant for large $y_{\mathrm{ijkr}}$ but approaches infinity as $y_{\mathrm{ijkr}}\to 0$. Log transformations are unsuitable for negative values, tend to be inflated for low values that are in the range of the background [17], and are not sensitive to other sources of MVD.

#### 2.4.2 Cyclic loess normalization

This stabilizes the MVD between slides by applying a pairwise non-linear local regression (LOESS). It utilizes a pseudo Bland-Altman (MA) plot defined as the average [A] versus the difference [M] between the intensities on two independent slides, repeated for *N* number of slides [29]. It yields an ‘average’ array that is used as a reference to adjust the MVD across all slides. It can be applied to both raw and log-transformed data [30]. Cyclic loess performs a pairwise normalization on all distinct pairs of slides utilising the MA plot and LOESS smoothing. The MA plot in single-colour microarrays for a pair of arrays is the scatter plot of average the intensity values [A] from both arrays vs. difference in expression values [M] of the same arrays The intensity-dependent differences are first estimated and the differences subsequently regulated by centering the LOESS line to zero [26], [29].

Given $\overset{⌢}{\underset{\mathrm{ijkr}}{y}}$ for a given slide i=1,2,3,.....,n,$M_{r}=\log_{2}\left(\overset{⌢}{\underset{1jkr}{y}}/\overset{⌢}{\underset{2jkr}{y}}\right)$ and $A_{r}=\frac{1}{2}\log_{2}\left(\overset{⌢}{\underset{1jkr}{y}}\times \overset{⌢}{\underset{2jkr}{y}}\right)$ where r=1,2,3,...,p are the spot intensities for a specific protein. A LOESS curve is then fitted for the MA differences and $M_{r}^{\prime }$ a normalised value for $M_{r}$ is generated. The spot for each specific protein intensities is normalized as follows ${\hat{y}}_{1jkr}^{\prime }=2^{A_{r}+\frac{M_{r}^{\prime }}{2}}$ and ${\hat{y}}_{2jkr}^{\prime }=2^{A_{r}-\frac{M_{r}^{\prime }}{2}}$ or the logarithm transformed equivalents [29], [31]. Here we use the LOESS method of Ballman et al. [8], [29].

The underlying assumption in cyclic loess is that there is minimal variation between individual arrays under the conditions being studied. Its application for protein microarray experiments designed to detect high levels of variation in different arrays may thus be limited. We recommend the randomization of samples during the design of the study to ensure there is minimal variation between the arrays.

#### 2.4.3 Robust linear normalization (RLM).

This method stabilizes the mean–variance dependence (MVD) by using standardized control spots on each slide to adjust intensities across the entire experiment [10]. It assumes that the signal detected from control spots remains constant with the exception of technical differences within or between slides. It is the method of choice when a significant amount of variation between samples is anticipated.

Data from control spots are fitted to a robust statistical model using an iteratively reweighted least-squares procedure with a robust “sandwich estimator”, like the median. Fixed effects for each array or slide and positive control proteins are estimated from the statistical model. Sboner et al. recommended using a linear model applied to log-transformed intensities [10]. The model $\log_{2}\left(\overset{⌢}{\underset{\mathrm{ijkr}}{y}}\right)=\alpha *{\text{Slide}}_{\text{i}}+\beta *{\text{Block}}_{\text{j}}+\tau *{\text{Protein}}_{k}+\varepsilon_{\mathrm{ijkr}}$, $\overset{⌢}{\underset{\mathrm{ijkr}}{y}}$ is the background-corrected intensity for the spot r of protein feature k in the block j on the $i^{\mathrm{th}}$ slide. α is the slide effect of the slide i, β is the block j effect and τis the effect of protein feature k ; this helps account for the spotted protein amount and binding affinity of different protein features and $\varepsilon_{\mathrm{ijkr}}=Norm\left(0,\sigma^{2}\right)$ is the error term. After estimating the best parameters, the transformed values are estimated as $\log_{2}\left({\hat{y}}_{\mathrm{ijkr}}^{\prime }\right)=\log_{2}\left({\hat{y}}_{\mathrm{ijkr}}^{\prime }\right)-\left(\alpha_{i}+\beta_{j}\right)$ [10]. We recommend that the control antigens used for normalization are optimized to avoid saturation to facilitate the identification of true technical variation. We used human Ig (IgG and IgM) as controls in our experiments.

As with other logarithm transformations, RLM is not suitable for negative signals values. These are consequently replaced using the moving minimum positive approach (above).

#### 2.4.4 Variance stabilization normalization (VSN)

The VSN method overcomes the limitations of log transformations by accommodating negative values and minimizing the inflated variance around low signal intensities. It calibrates between-feature variation through shifting and scaling mechanism in which all the data are adjusted.

Huber et al. and Durbin et al. independently proposed the VSN approach which is a variant of the log-transform (*glog2*). A two-component model to explain the proportional increase in the variance with the mean intensity of the proteins was proposed [9], [17], [27]; $y_{\mathrm{ijkr}}=\overset{⌢}{\underset{\mathrm{ijkr}}{\alpha }}+\mu_{\mathrm{ijkr}}e^{\eta }+\varepsilon_{\mathrm{ijkr}}$, where $\overset{⌢}{\underset{\mathrm{ijkr}}{\alpha }}$ is the background signal and $\mu_{\mathrm{ijkr}}=\overset{⌢}{\underset{\mathrm{ijkr}}{y}}$ is the actual signal. $\eta =Norm\left(0,\sigma_{\eta }\right)$ and $\varepsilon_{\mathrm{ijkr}}=Norm\left(0,\sigma^{2}\right)$ are the proportional error and background error respectively. However, with background corrected data this can be modelled as $y_{\mathrm{ijkr}}\approx \mu_{\mathrm{ijkr}}e^{\eta }$. A transformation h is used to produce values such that $Var\left(h\left(y_{\mathrm{ijkr}}\right)\right)$ is approximately independent of the mean, $E\left(h\left(y_{\mathrm{ijkr}}\right)\right)$. In general, for a matrix, $\mu_{\mathrm{ijkr}}$ the function implemented fits a normalisation transformation $\mu_{\mathrm{ijkr}}\to h\left(\mu_{\mathrm{ijkr}}\right)={\text{glog}}_{2}\left(\frac{\mu_{\mathrm{ijkr}}-a_{i}}{b_{i}}\right)$ where $b_{i}$ is the scaling parameter for array i which is always ensured to be positive with a parameter transformation f(b)=exp(b), $a_{i}$ is the background offset included if the data is not background corrected and $\mathrm{glo}g_{2}\left(u\right)=\log_{2}\left(u+\sqrt{u^{2}+1}\right)=\text{arsinh}\left(u\right)/\log\left(2\right)$ is the generalised transformation h. A robust variant of the maximum likelihood estimator for the 2 parameters is utilised [1]. Each slide is treated independently and slide to slide variation is not considered [10].

### 2.5 Batch Correction.

The processing of samples on separate days introduces batch-to-batch variations due to non-specific day to day differences in laboratory conditions or operators [18].

We implement a selection of tools to identify and visualize batch effects such as coloured scatter plots, hierarchical clustering or principal component analysis (PCA). Subsequent analyses can be adjusted to account for batch effects, but the majority are designed for large experiments of at least 25 batches [32]. We utilise the Empirical Bayes approach as it accommodates all batch sizes and can utilize both parametric and non-parametric data [18].

#### 2.5.1 Empirical Bayes (EB) batch correction using ComBat

This uses the EB approach to estimate and correct batch effects. It can be applied to high-dimensional data even when the sample size is small. Suppose we have b batches in the data containing $n_{s}$ samples within a batch w for w=1,2,....,b and a protein k=1,....,K then a location and scale (L/S) adjustment model is assumed;$Y_{\mathrm{wsk}}=\alpha_{k}+X\beta_{k}+\gamma_{\mathrm{wk}}+\delta_{\mathrm{wk}}\varepsilon_{\mathrm{wsk}}$. Then, the EB batch adjusted data $\gamma_{\mathrm{wsk}}^{*}$ is then calculated as follows $\gamma_{\mathrm{wsk}}^{*}=\frac{{\hat{\delta }}_{k}}{{\hat{\delta }}_{\mathrm{wk}}^{*}}\left(Z_{\mathrm{wsk}}-{\hat{\gamma }}_{\mathrm{wk}}^{*}\right)+{\hat{\alpha }}_{k}+X{\hat{\beta }}_{k}$[18] [Supplementary B for details]. To perform this, we utilise a wrapper to *SVA’s* function *ComBat()* for the batch adjustment that has both the parametric and non-parametric approaches [18].

## 3 Results

### 3.1 Implementation

protGear is an R based suite with a range of functions to facilitate protein microarray data pre-processing. It has a built-in user-friendly Shiny® dashboard [Supplementary C1 Fig. 1 and Supplementary C2] to assist in real-time processing, visualization and downstream analysis using heatmaps and Principal Component Analysis (PCA). It provides five sequential steps for handling a data table of fluorescent intensities. Importantly, the package enables the inclusion of additional functions that may be deemed useful. A detailed workflow is included in the *protGear_vignette* document in the supplementary or https://keniajin.github.io/protGear/.

### 3.2 Background correction

**Fig. 3:**
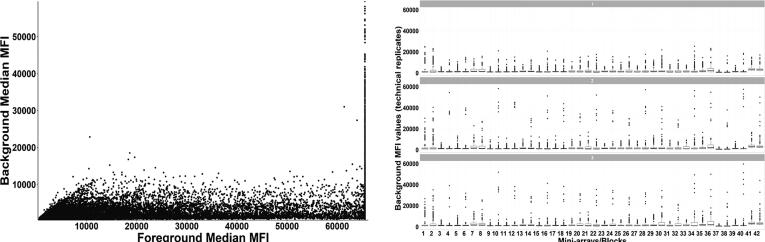
Example of background diagnostic plots produced by protGear. (A) is the background MFI vs foreground MFI plot that is useful to assist in selecting the appropriate background correction method. (B) is a boxplot of the blocks/mini arrays categorised into the technical repeats. This plot is important to check whether there is a block artefact in the background MFI values.

The protGear *background_correct* function implements five different techniques for background correction that are complemented by diagnostic plots. Fig. 3 shows the example of a background diagnostic plot produced by protGear. As shown in Fig. 3 similar local background values were observed across the different blocks. The correlation between the foreground and background intensities (medians) on the same array was also low. Therefore, a local background correction approach was selected and applied.

### 3.3 Within sample variation and purification tag subtraction

**Fig. 4:**
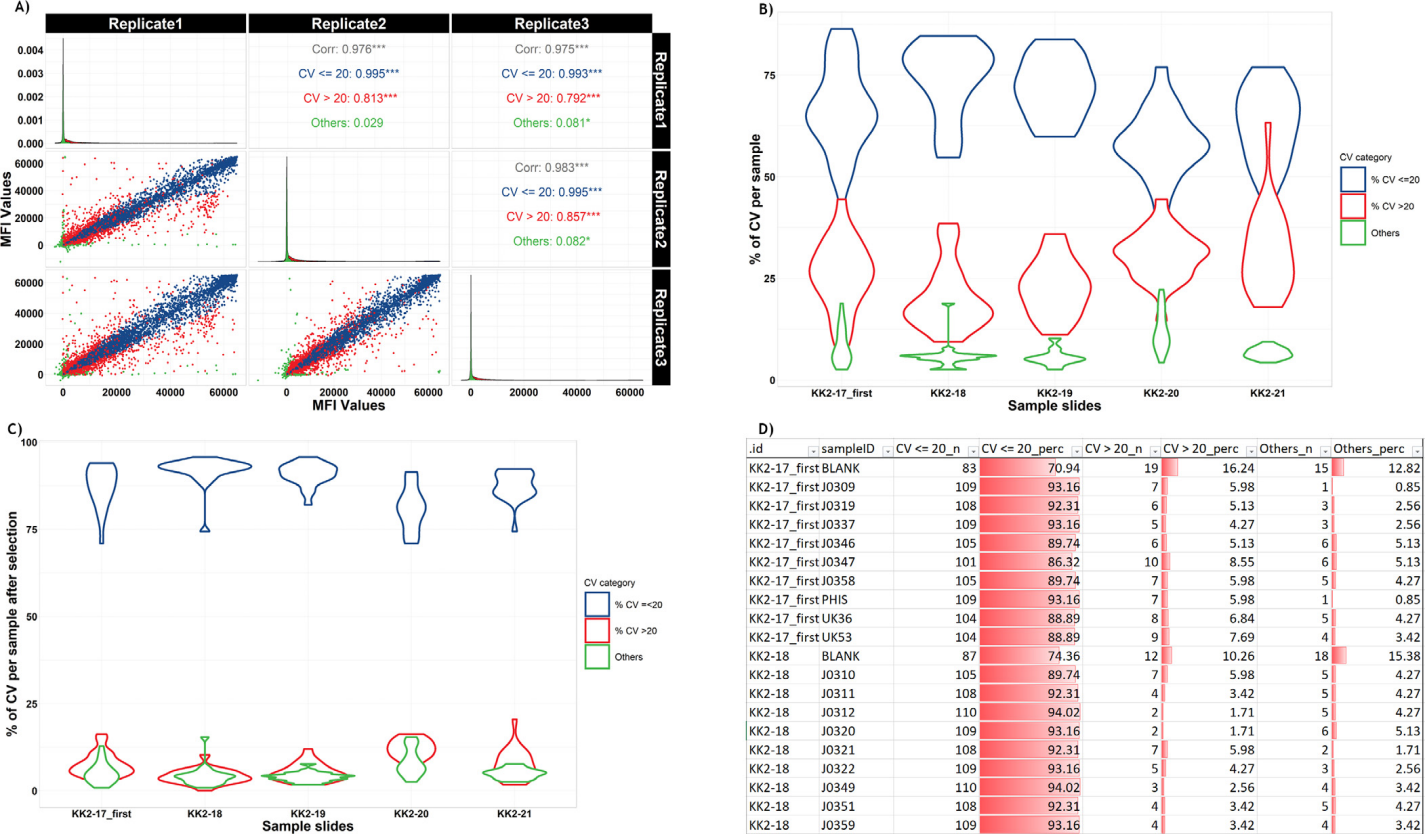
The visualization of the CV A) Correlation of the technical replicates, B) Proportion of CV by the CV cut off, C) Proportion of CV after “cv_based_filtering”, D) A static image of an Interactive table to inspect the CV cut off values. The table shows the specific slide id (.id), the serum sample identifier (sampleID), count of CV’s < 20% (CV<=20), % of CV’s < 20%, count of CV’s > 20% (CV > 20), % of CV’s > 20% and out of range CVs on the 1st to 8th columns, respectively.

protGear provides a *cv_estimation* function that is applied to technical repeats to calculate and visualize within-sample variability utilizing a user-defined CV. A filtering algorithm identifies technical repeat spots meeting user-defined criteria e.g. CV < 20%. The function generates a flag variable to enable further scrutiny of non-performing spots. Fig. 4 illustrates graphics to monitor the CV before and after filtering. A folder is created to store the CV-corrected data in the working directory or the package function, respectively. An average of the replicates is calculated before the subtraction of the intensity measured against the purification tag (optional). Here, we kept 2 of 3 technical replicates with CVs<20% and excluded the outlier value.

### 3.4 Normalisation and batch correction

**Fig. 5:**
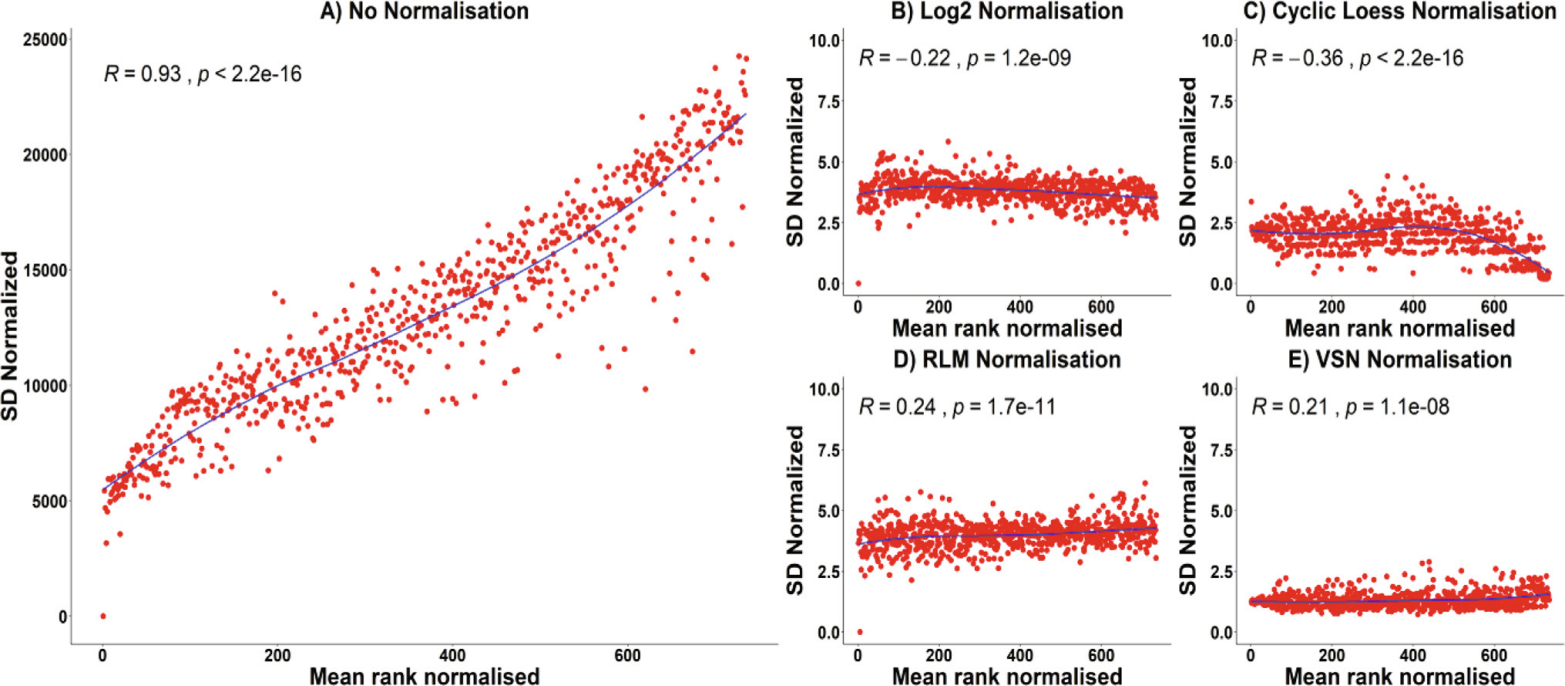
Standard deviation vs mean plots (meanSdPlot) of A) Non normalised data B) log2 normalisation C) Cyclic loess normalisation D) Robust Linear Model normalisation and E) VSN normalisation.

We tested four functions for normalization to identify the one that optimally reduced the MVD. We formally assessed this using the mean versus standard deviation plots (*meanSdPlots*) and coupled this to automatically derived Spearman correlation estimates (*Rho*) and Cox–Stuart or the Mann–Kendall trend tests. The latter quantifies the performance of the normalization. In the example below, we illustrate the versatility of the tool across four Methods for normalization. Using these approaches, the MVD reduction led to a drop in the Rho estimate from 0.93 to between 0.2 and 0.3 in this dataset as shown in Fig. 5 below. Implementation of the log_2_ approach led to an inflation of variance for the low MFI (Mean Fluorescent Intensity) values. The cyclic loess and

RLM were not optimal for the experimental design (discussed in the methods). Consequently, the VSN normalization approach was adopted (Fig. 5). We then proceeded to investigate batch effects using *ComBat* from the SVA package*.* The day-to-day dependence was noticeably reduced by *ComBat* batch correction [Supplementary B Fig. 1] for day m1 and m2.

## 4 Discussion

protGear is an open-source one-stop integrated data pre-processing suite specifically tailored to address the systematic and non-systematic sources of bias in high throughput protein microarray experiments. It can be adapted to a range of design formats and is compatible with the majority of commercial protein microarray platforms. It provides a choice of functions to address each major source of bias, outlines the theoretical background necessary to guide selection and generates custom graphical and tabular outputs to support data interrogation and interpretation. It is coupled with a user-friendly interactive web platform that eliminates the need for specialized programming skills. In line with other open-source platforms, protGear can be adapted to incorporate new functions either by adding custom scripts or contributing to its online repository.

The localization and distribution of background signal intensities guide the selection of the appropriate correction strategy [16], [21], [22]. Our experimental design comprised multiple mini-arrays and slides. We did not detect any significant background pattern within the mini-arrays or block and consequently selected the local background correction method. Although unmodified background intensities are subtracted from the foreground resulting in unbiased estimates [16], negative values are often generated, and the consistency of the results across different mini-arrays and slides needs to be assured. Additional methods including the novel *half moving minimum* we developed that overcome these limitations have been included [8].

The inclusion of two or more technical replicates is vital for the robust analysis of within-sample variability [15]. Caution in interpreting the CV is recommended as small differences in signal intensities between replicates at the lower end of the detection threshold can yield misleadingly high CVs. To overcome this, we implemented a novel function that enables users to set signal intensity thresholds below which high CVs can be ignored.

The importance of adopting an appropriate strategy for normalization is critical and requires the coupling of the experimental hypothesis with the mathematical assumptions underpinning the methods [33]. Key design issues to consider are the extent of sample biological variation anticipated under the conditions being investigated and technical considerations in the array design such as the inclusion of appropriate controls. Adopting the wrong method increases the probability of detecting false-negative and false positives.

The RLM normalisation approach that was specifically developed for protein microarrays [10] requires that the secondary control normalisation proteins yield constant signals across and within slides. These control proteins are expected to have a low CV and can be used for both normalizations and evaluating the slide variability [10]. Although the MVD was significantly reduced with all methods tested, the VSN method was optimal. protGear provides a novel powerful single platform that empowers users to sequentially interrogate all these options to determine the optimal solution for the data in question. The easy-to-use interface accommodates multiple data formats, saves enormous amounts of time and generates high-quality visual outputs that facilitate rapid decision making.

Batch effects create an additional unwelcome source of variation that could further reduce statistical power. These must be considered when data are processed at different times, by different users and on different instruments, among others [34]. An important consideration that particularly applies to large cohort studies is the random processing of samples to ensure that batch effects do not exaggerate pre-existing genuine biological variation. For example, in the context of malaria seroepidemiology, age and geographical location are critical determinants of antibody levels [35]. The processing of samples of young children at one time-point, and those of older children at another, could inadvertently lead to enhanced differences in either group that were unrelated to the true underlying biological variation. A similar effect could occur when samples from settings with differing malaria transmission intensity are analysed in the same experiment but at separate times.

A limited number of batch correction approaches have been proposed and these typically accommodate experimental designs with a minimum of 25 batches. We adopted the ComBat batch correction function from the “sva” package in R since it has been reported to be robust to outliers in small batch sizes [18].

protGear provides a state-of-the-art, one-stop adaptable workflow for protein microarray data pre-processing. It can be coupled to software such as Sweave or knitr [36] for report generation and sharing along with the raw data to promote data reproducibility. The user-friendly, interactive, web-based and graphical interface requires limited R-experience and will enhance broad uptake in the infectious disease and vaccine development community.

## 5 Availability and implementation

The protGear R package is publicly available in the GitHub repository (https://github.com/Keniajin/protGear).

## Funding

This research was commissioned by the National Institute for Health Research (NIHR) Global Health Research programme (16/136/33) using UK aid from the UK Government. The views expressed in this publication are those of the author(s) and not necessarily those of the NIHR or the Department of Health and Social Care.

K.M, F.O, S.K are funded by NIHR Global Health Research Unit Tackling Infections to Benefit Africa (TIBA). F.O. is supported by a Sofja Kovalevskaja Award from the Alexander von Humboldt Foundation (3.2–1184811 - KEN - SKP) and an EDCTP Senior Fellowship (TMA 2015 SF1001) which is part of the EDCTP2 Programme supported by the European Union. S.K and E.M are supported by the DELTAS Africa Initiative under Initiative to Develop African Research Leaders (IDeAL) and DELTAS Sub-Saharan Africa Consortium for Advanced Biostatistics (SSACAB) respectively.

## CRediT authorship contribution statement

**Kennedy Mwai:** Conceptualization, Methodology, Software, Writing - original draft, Writing - review & editing, Visualization. **Nelson Kibinge:** Conceptualization, Methodology, Software, Writing - original draft, Supervision, Writing - review & editing, Visualization. **James Tuju:** Conceptualization, Writing - original draft, Investigation, Supervision, Writing - review & editing. **Gathoni Kamuyu:** Literature search, Writing - original draft, Investigation, Writing - review & editing. **Rinter Kimathi:** Data curation, Literature search, Investigation. **James Mburu:** Methodology, Software, Visualization. **Emily Chepsat:** Data curation, Investigation. **Lydia Nyamako:** Data curation, Investigation, Project administration. **Timothy Chege:** Data curation, Investigation. **Irene Nkumama:** Data curation, Investigation. **Samson Kinyanjui:** Supervision, Funding acquisition, Writing - review & editing. **Eustasius Musenge:** Conceptualization, Methodology, Writing - original draft, Supervision, Writing - review & editing. **Faith Osier:** Conceptualization, Methodology, Writing - original draft, Supervision, Funding acquisition, Writing - review & editing.

## Declaration of Competing Interest

The authors declare that they have no known competing financial interests or personal relationships that could have appeared to influence the work reported in this paper.
