# Supplementary material for: protGear: A protein microarray data pre-processing suite
Source: Comput Struct Biotechnol J. 2021 Apr 24;19:2518–25. doi: 10.1016/j.csbj.2021.04.044 (PMC8114118; doi:10.1016/j.csbj.2021.04.044)
Supplement: Supplementary Data 2 [file mmc2.pdf]

Supplementary A - Definition of terms and quantitation results file contents

16 April, 2021

Quantification results file contents

A quantification results file contains the data for multiple parameters quantified and measured in relation to a given spot. Importantly, it has the location details of each spot as defined in the input file. Below we show examples of parameters generated by the GenePix^R Pro software contained in the genepix results file (.gpr). The first part is the header which describes basic file information and all block properties. Importantly, it has the location details of each spot, the row (Y coordinate) and column (X coordinate).

```
ATF 1.0
30  41
"Type=GenePix Export 3"
"BlockCount=4"
"DateTime=2017/08/29 13:39:04"
"Settings="
"GalFile=\Multicenter study\gal file\gal file_4`.gal"
"PixelSize=10"
"Wavelengths=635"
"ImageFiles=Z:\Multicenter study\28_08-2017\datascan_28-8\KK2-07.tif 0"
"NormalizationMethod=None"
"NormalizationFactors=1"
"JpegImage="
"StdDev=Type 1"
"RatioFormulations=W1/W2 (635/594)"
"FeatureType=Circular"
"Barcode="
"BackgroundSubtraction=LocalFeature"
"ImageOrigin=0, 0"
"JpegOrigin=1740, 900"
"Creator=GenePix Pro 7.3.0.0"
"Scanner=GenePix 4300 [141110]"
"FocusPosition=0"
"Temperature=0"
"LinesAveraged=1"
"Comment="
"PMTGain=400"
"ScanPower=100"
"LaserPower=0.66"
"Filters=Standard Red"
"ScanRegion=0,0,2200,7200"
"ScanArea=1"
"Supplier="
"Flags" "Normalize" "Autoflag"  "Block" "Column"    "Row"   "Name"  "ID"    "X" "Y" "Dia."  "F635 Median"
0   0   0   1   1   1   Landmark    1F1 2310    1130    150 65535
0   0   0   1   2   1   MSP3.6  1B3 2540    1130    150 13182
0   0   0   1   3   1   ETRAMP10.2  1H3 2770    1130    150 2310
0   0   0   1   4   1   GAMA    1D5 3000    1130    150 18395
```

- **Block** - the block number of the feature. The array has a number of blocks. For example GenePix assigns block numbers row wise from left to right. The top left block is assigned block #1 as shown below.
- **Column** - the column number of the feature.
- **Row** - the row number of the feature/protein.

1	2	3
4	5	6
7	8	9
10	11	12
13	14	15
16	17	18
19	20	21

Table 1: Block numbering og GenePix

- **Name** - the name of the feature/protein derived from the Array List (up to 40 characters long, contained in quotation marks).
- **ID** - the unique identifier of the feature/protein derived from the Array List (up to 40 characters long, contained in quotation marks).
- **X** - the X-coordinate in nm of the center of the feature/protein-indicator associated with the feature/protein, where (0,0) is the top left of the image.
- **Y** - the Y-coordinate in nm of the center of the feature/protein-indicator associated with the feature/protein, where (0,0) is the top left of the image.
- **Dia.** - the diameter in *nm* of the feature/protein-indicator.
- **F635 Median** - median feature/protein pixel intensity at wavelength (635 nm). This changes according to the wavelength used to scan.
- **"B635 Median"** - background pixel intensity at wavelength (635 *nm*). This changes according to the wavelength used to scan.
- **Flags*** - the type of flag associated with a feature/protein.

Definition of terms

- **Scanner:** an instrument used to detect and quantify the intensity of fluorescence of spots on a micro-array slide.
- **Mini-array** - a number of spots on the array representing data for a given sample.
- **Block** - a collection of spots within a mini-array. A number of blocks can form a mini-array depending on the design of the experiment.
- **Pixel** - Pixels are small geometrical digital subunits with a corresponding fluorescence estimated as a number during the quantification process. A number of pixels form a spot.
- **A spot** - it is a measure of resolution differing from the surface around it made up of a number of pixels. The brighter the spot is the higher the scanning value. The fluorescence is averaged from the pixels that make up the spot.
- **Sample ID file** - A file with names of samples with their corresponding blocks as they were arranged on the slide.
