# Supplementary material for: protGear: A protein microarray data pre-processing suite
Source: Comput Struct Biotechnol J. 2021 Apr 24;19:2518–25. doi: 10.1016/j.csbj.2021.04.044 (PMC8114118; doi:10.1016/j.csbj.2021.04.044)
Supplement: Supplementary Data 3 [file mmc3.docx]

**ComBat Batch Correction**

The processing of samples on separate days or in different sets on the same day introduces batch-to-batch variations due to differences in laboratory conditions or in operators (1,2). ssHere we show how the evaluation of EB batch adjusted data $\gamma_{wsk}^{*}$ is calculated from (2)

Application: Suppose we have $b$ batches in the data containing $n_{s}$samples within batch $w$ for $w=1,2,....,b$ and a protein $k=1,....,K$ then a location and scale (L/S) adjustment model is assumed;$Y_{wsk}=\alpha_{k}+X\beta_{k}+\gamma_{wk}+\delta_{wk}\varepsilon_{wsk}$ and that the errors $\varepsilon_{wsk}\sim Norm\left(0,\delta_{k}^{2}\right)$. The above model is defined with non-standardized data and this implies that $\alpha_{k},\beta_{k},\gamma_{k}\text{ and }\delta_{k}^{2}$ differ across proteins (2). This can bias the estimates of EB estimates of the prior distribution of batch effect and reduce the amount of systematic batch information that can be borrowed across proteins. However, this can be avoided by standardizing the data or applying a non-parametric approach (3).

Assuming, standardized data $Z_{wsk}\sim Norm\left(\gamma_{wk},\delta_{wk}^{2}\right)$ with parametric forms for the prior distribution of the batch effects $\gamma_{wk}\sim Norm\left(Y_{w},\tau_{w}^{2}\right)$ and the batch effect parameters, $\gamma_{wk}$and $\delta_{wk}^{2}$are estimated by the conditional posterior means (2). The EB batch adjusted data $\gamma_{wsk}^{*}$ is then calculated as follows (2).To perform this, we utilise a wrapper to SVA’s function ComBat() for the batch adjustment that has both the parametric and non-parametric approaches (3).

Figure : Comparison of Batch m1 and Batch m2 before and after batch correction using Combat() approach. The image on the left shows the MFI rank vs MFI values before batch correction for batch m1 and m2 and the image on the right shows the same MFI values after batch correction.

**References:**


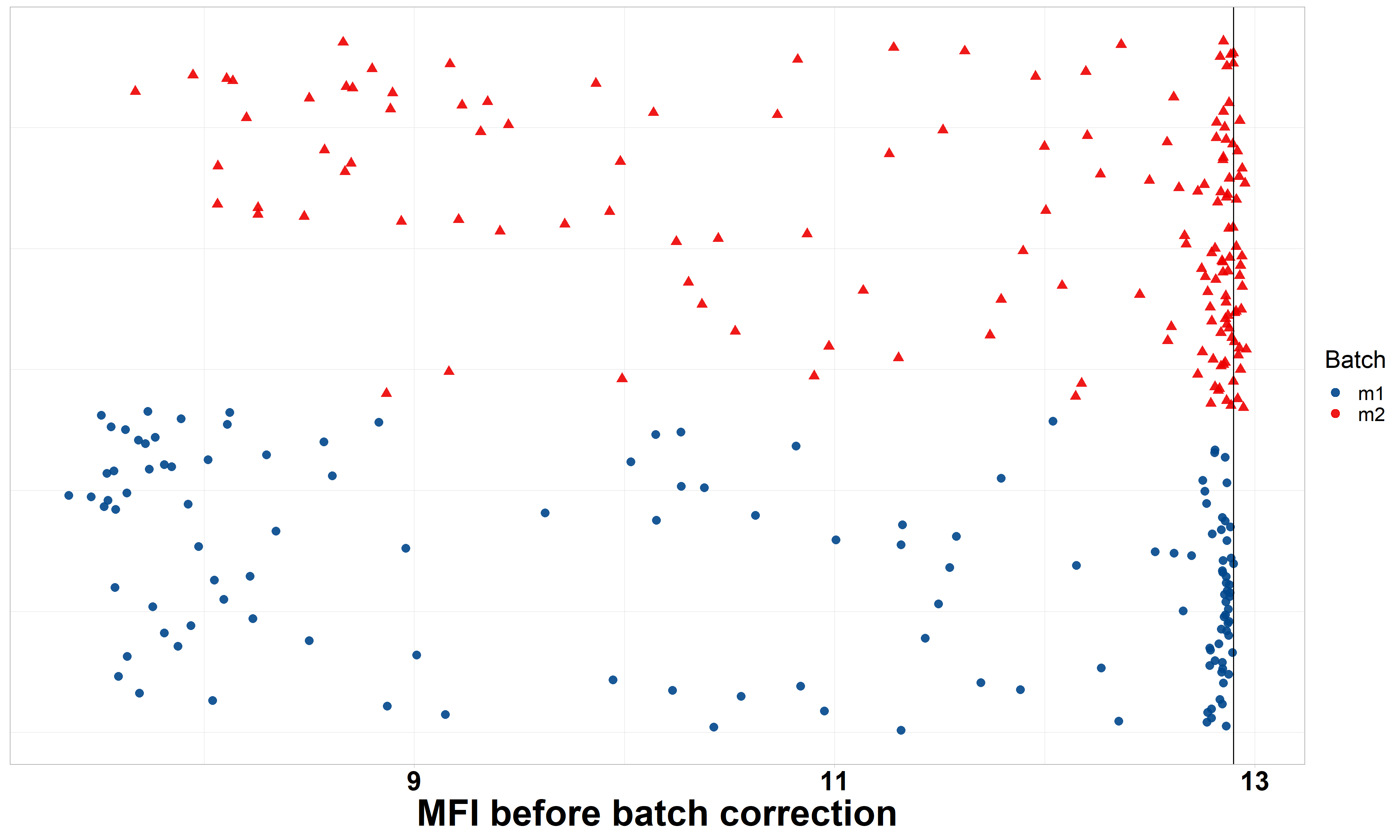

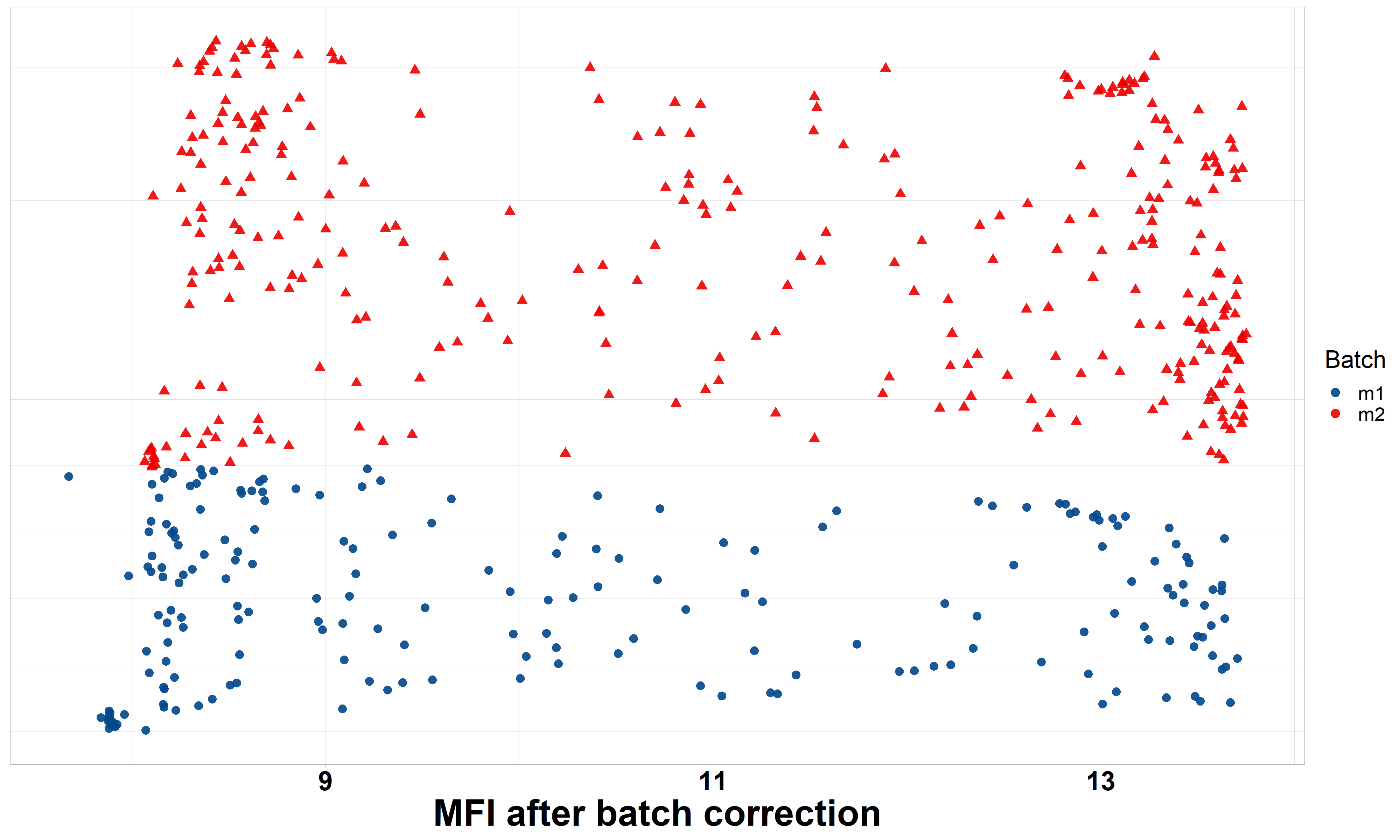


**MFI Rank**

1. Yuan J, Hegde PS, Clynes R, Foukas PG, Harari A, Kleen TO, et al. Novel technologies and emerging biomarkers for personalized cancer immunotherapy. J Immunother cancer. 2016;4(1):3.

2. Johnson WE, Li C, Rabinovic A. Adjusting batch effects in microarray expression data using empirical Bayes methods. Biostatistics. 2007;8(1):118–27.

3. Leek JT, Johnson WE, Parker HS, Jaffe AE, Storey JD. The sva package for removing batch effects and other unwanted variation in high-throughput experiments. Bioinformatics. 2012;28(6):882–3.
