# Supplementary material for: protGear: A protein microarray data pre-processing suite
Source: Comput Struct Biotechnol J. 2021 Apr 24;19:2518–25. doi: 10.1016/j.csbj.2021.04.044 (PMC8114118; doi:10.1016/j.csbj.2021.04.044)
Supplement: Supplementary Data 4 [file mmc4.pdf]

	Comparison between currently available protein microarray analysis tools						
	**protGear**	**Protein Microarray Analyser (PMA)**	**Protein Array Analyzer (PAA)**	**protein array web exploreR (PAWER)**	**RPPanalyzer**	**RPPASPACE**	**protein microarray database (PMD)**
**Language**	R and Shiny	Java.	R	R and JavaScript	R	R	PhP and R
**Source code contribution**	✓	X	X	✓	✓	✓	X
**Vignettes: long-form documentation**	✓	X	✓	X	X	✓	X
**Interactive GUI**	✓	✓	X	✓	X	X	✓
**Mutiple methods comparison**	✓	X	X	X	X	X	X
**Background corection**	Local and Global Background, moving minimum, half moving minimum, normexp and log linear	Local Background Subtraction	Not specified	Local Background Subtraction	Limma background correct and addmin	Local background subtraction	Not Clear
**Normalization**	Log, RLM, VSN and Cycli Loess	Pin to Pin and Array to Array Normalization	RLM	RLM	proteinDye,row, housekeeping and extValue	spatial corrections	Not Clear
**Downstream Analysis**		X	✓	X	X	X	✓
**Accessibility**	✓	https://sourceforge.net/proje cts/protein-micro array-analyser/	https://bioconductor.org/packages/release/bioc/html/PAA.html	https://biit.cs.ut.ee/pawer/	https://cran.r-project.org/web/packages/RPPanalyzer/index.html	https://cran.r-project.org/web/packages/RPPASPACE/index.html	http://www.proteinmicroarray.cn/
**License**	GPL-3	No License Specified	3-Clause BSD License	GNUGPL v2 license	LGPL-3	Artistic License 2.0	Creative Commons Attribution 4.0 International
**Last updated**	2021	2018	2020	2020	2020	2020	2021
