# Supplementary material for: protGear: A protein microarray data pre-processing suite
Source: Comput Struct Biotechnol J. 2021 Apr 24;19:2518–25. doi: 10.1016/j.csbj.2021.04.044 (PMC8114118; doi:10.1016/j.csbj.2021.04.044)
Supplement: Supplementary Data 4 [file mmc5.pptx]

## Slide 1
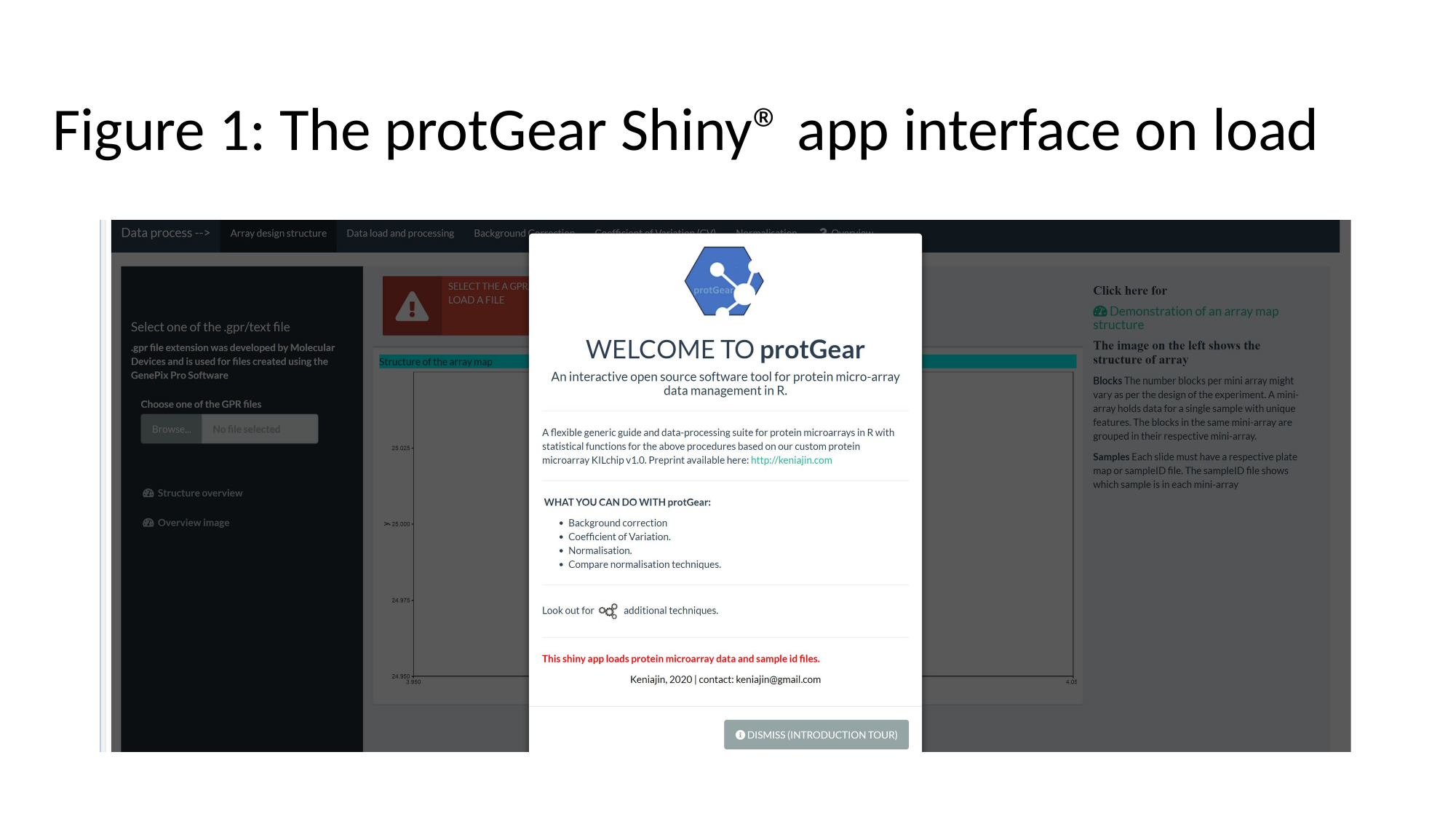

# Figure 1: The protGear Shiny® app interface on load

## Slide 2
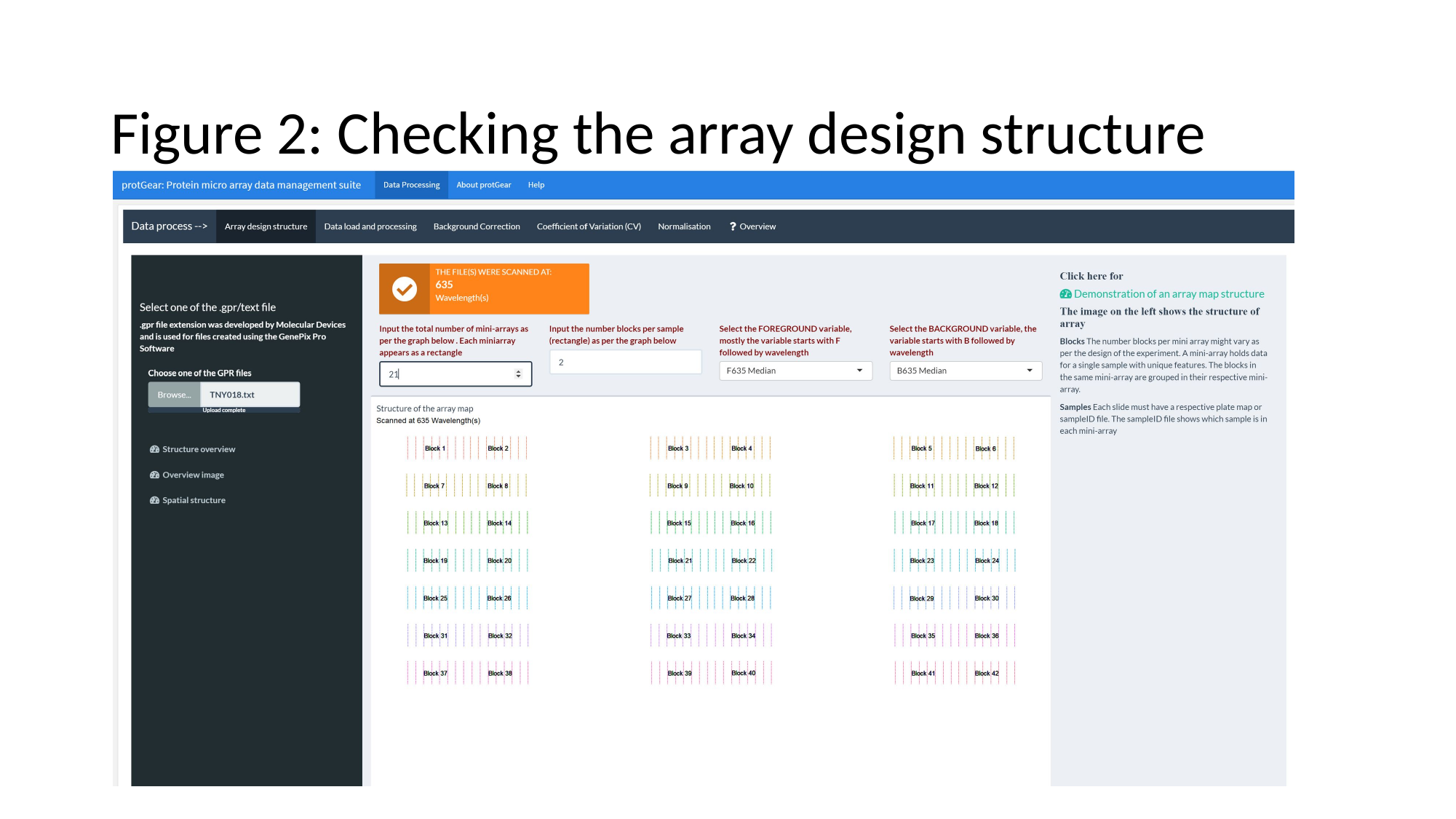

# Figure 2: Checking the array design structure

## Slide 3
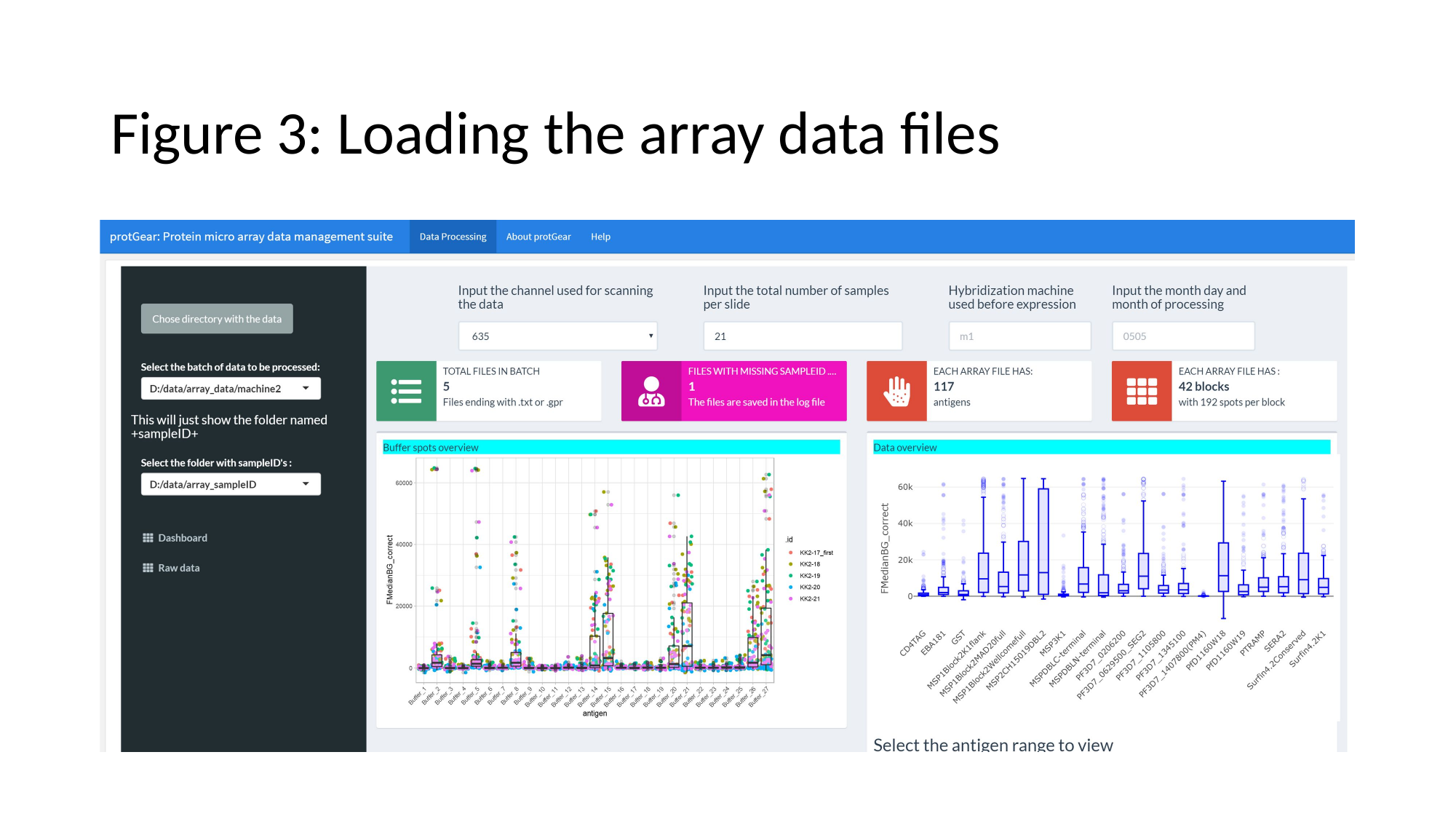

# Figure 3: Loading the array data files

## Slide 4
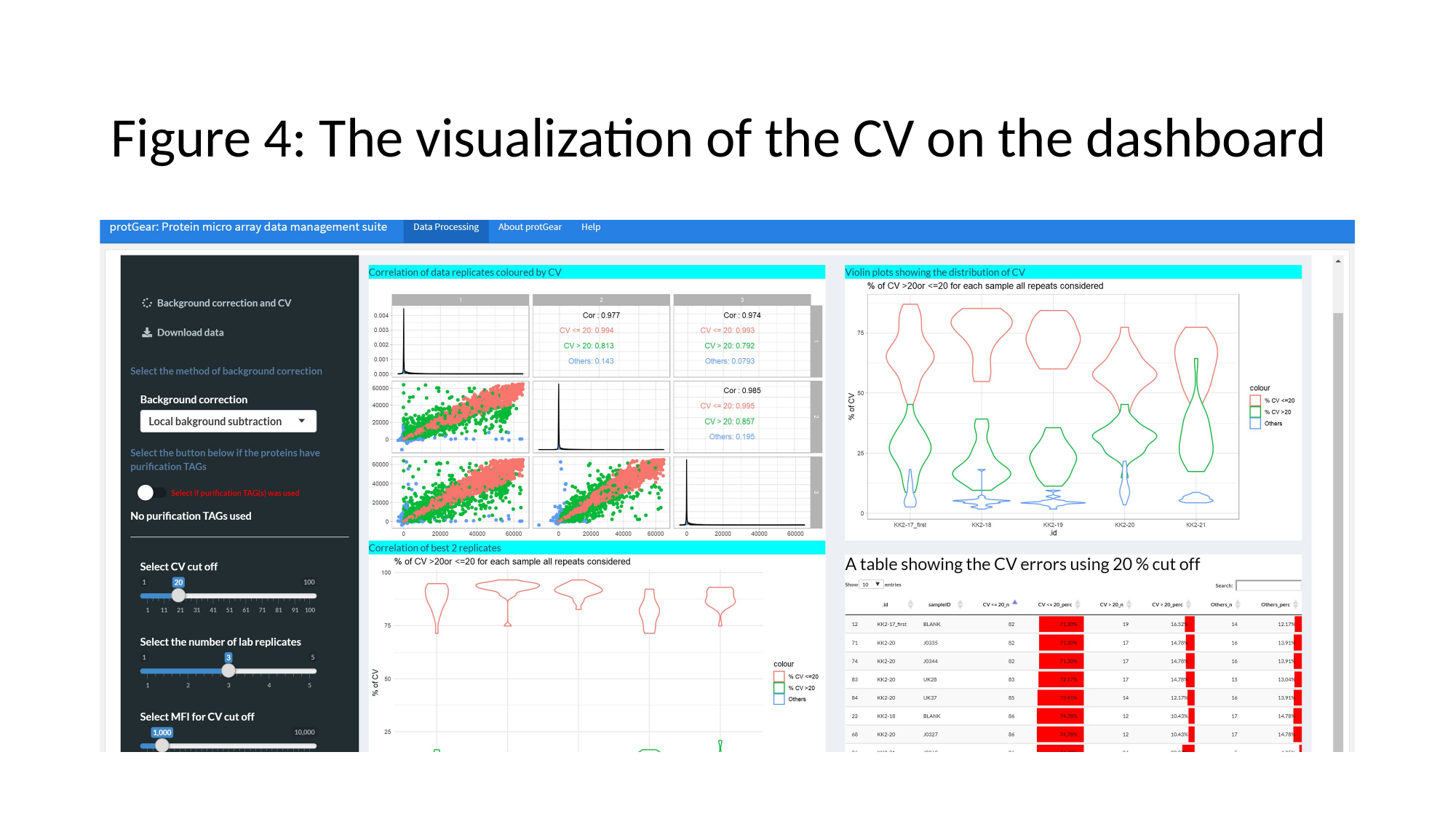

Figure 4: The visualization of the CV on the dashboard

## Slide 5
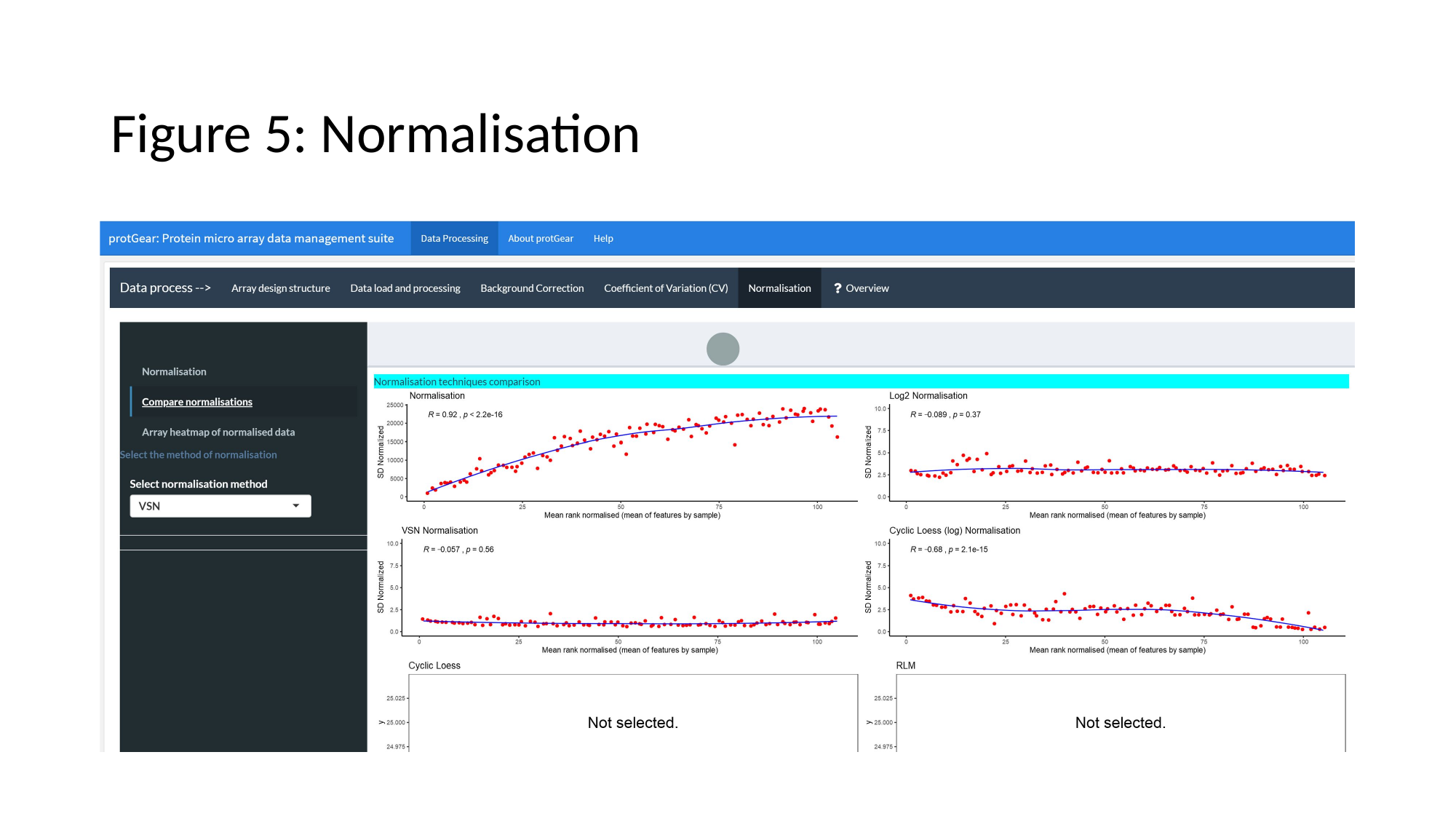

# Figure 5: Normalisation

## Slide 6
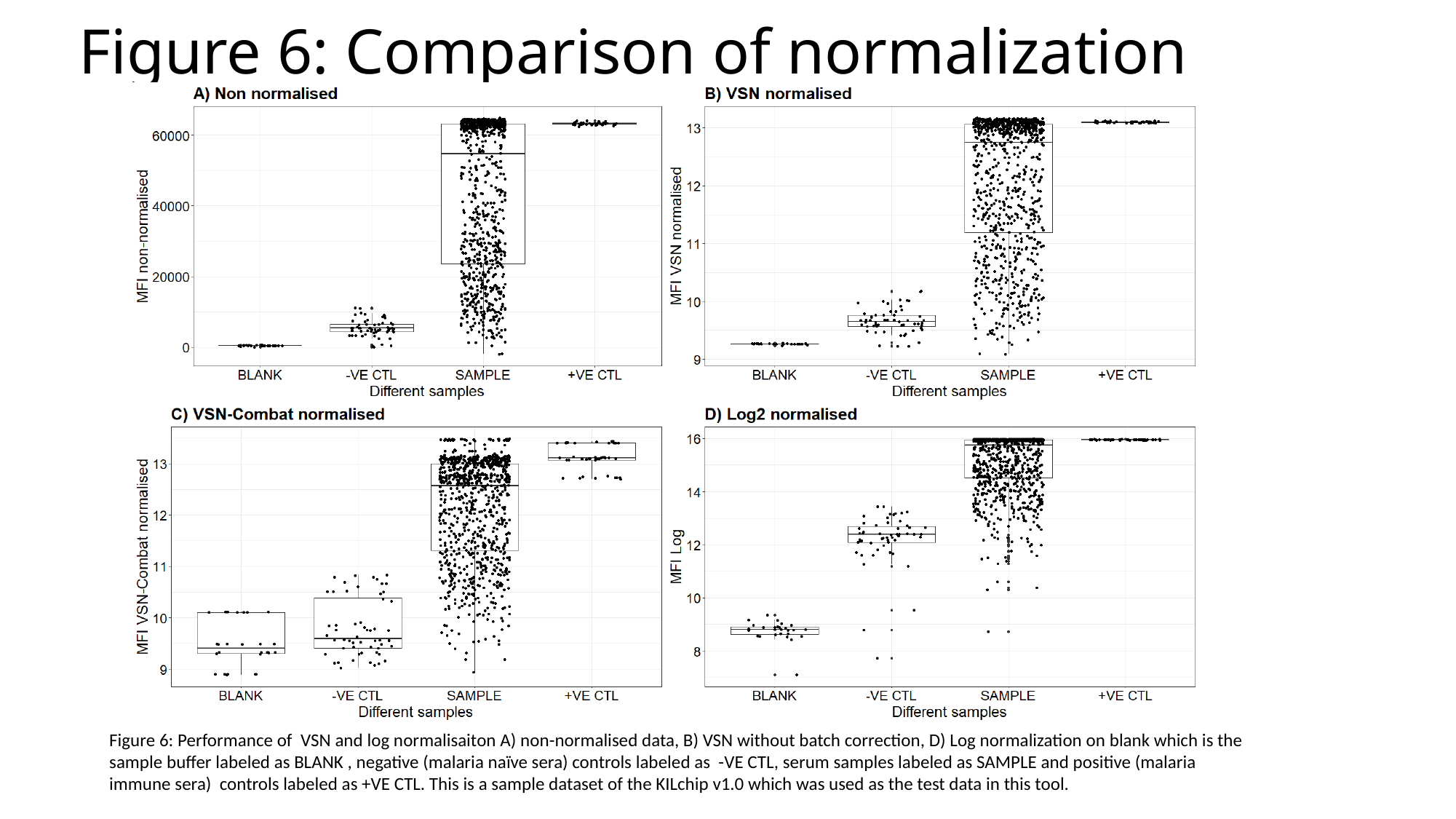

# Figure 6: Comparison of normalization
Figure 6: Performance of VSN and log normalisaiton A) non-normalised data, B) VSN without batch correction, D) Log normalization on blank which is the sample buffer labeled as BLANK , negative (malaria naïve sera) controls labeled as -VE CTL, serum samples labeled as SAMPLE and positive (malaria immune sera) controls labeled as +VE CTL. This is a sample dataset of the KILchip v1.0 which was used as the test data in this tool.
